# The rise of mobile apps for the management of chronic liver disease

**DOI:** 10.1097/HC9.0000000000000429

**Published:** 2024-04-03

**Authors:** N. Begum Ozturk, Elliot B. Tapper

**Affiliations:** 1 Department of Internal Medicine, Beaumont Hospital, Royal Oak, Michigan, USA; 2 Division of Gastroenterology and Hepatology, Department of Internal Medicine, University of Michigan, Ann Arbor, Michigan, USA

Chronic liver disease (CLD) is common, with increasing incidence, morbidity, and mortality. The optimal management of CLD is defined by prevention in its earlier stages—through lifestyle modification (weight loss and exercise or alcohol avoidance)—and, in its later stages, the careful implementation of treatments with narrow therapeutic windows, such as diuretics and lactulose. Although outpatient visits are essential to educate, assess, and monitor CLD, the frequency of visits is neither sufficient to reinforce behavior change nor capture decompensation events early enough to reliably avoid hospitalization. Mobile apps are a powerful tool that could extend the impact of subspecialty care through telehealth, remote monitoring, and facilitated self-efficacy to improve the management of complications in patients with CLD.

## THE MOBILE APP ERA

Mobile apps have the potential to be an adjunct to close outpatient monitoring of signs or symptoms of the complications of CLD and provide reminders (for labs or medications). Mobile apps may increase access to care teams, particularly for those with transportation insecurity and inflexible work schedules. Several apps have been developed and studied in patients with CLD to assess medication adherence, monitoring of weight, vital signs, diet, and cognitive function, and to provide education on self-management (Table 1).

**TABLE 1:** Summary of mobile apps for chronic liver disease since 2020

References	Participants	Aim	Intervention	Findings
Sordi Chara et al^1^ Dieta app Prospective, feasibility trial	22 patients with cirrhosis and HE on lactulose	To evaluate patient experience with Dieta app To achieve BM and BSS goal (2–4 BM/day with BSS types 3-5)	Collection of stool images and weekly surveys of perceived HE-related symptoms and BM goal achievement Lead-in phase: No app feedback Intervention phase: Lactulose dose recommendations through app	High compliance and positive overall experience with the app (≥85% image uploading rate, 95% of participants would recommend the app to others, 64% felt their symptoms were better controlled). Lead-in phase: BSS goal achieved in 68% of the days with 74% survey completion rate. Absence of self-perceived confusion: 79%. Absence of sleep issues: 41%. Intervention phase: BSS goal achieved in 74% of the days with 84% survey completion rate. Absence of self-perceived confusion:75%. Absence of sleep issues: 51%. 4 patients were hospitalized during the study, only 1 for overt HE.
Fagan et al^2^ Dieta app Prospective, observational	First phase: 35 patients with cirrhosis and 13 controls Second phase: 8 patients with cirrhosis and 4 controls	To determine the utility of the Dieta app to classify BSS (AI-BSS) vs self-assessment (self-BSS) in longitudinal experiences	Collection of stool images/date/time First phase: AI-BSS communication is on Second phase: AI-BSS communication is off	First phase: AI-BSS significantly correlated with self-BSS and with daily BMs. Self-BSS was not correlated with daily BMs. Titration of the lactulose dose shifted from using BMs/day to AI-BSS by days 7 and 10. Second phase: Lactulose titration shifted from BMs/day towards BSS due to app feedback.
Wu et al^3^ AWARE app Prospective, observational	24 patients with ALD and AUD	To assess feasibility of the digital phenotyping of patients with ALD and AUD To determine correlations between smartphone sensor data and alcohol craving	Continuous smartphone hardware and software sensor data for EMAs	50% retention rate during the study period. Alcohol craving significantly correlated with mood obtained from EMAs. Significant relationships between craving, and features of location entropy and average accelerometer magnitude.
Sato et al^4^ NASH app Multicenter, single-arm trial	19 patients with biopsy-confirmed MASH	To assess the clinical efficacy of digital therapeutics in patients with MASH	App-provided counseling sessions, educational videos, advice on specific action goals to improve lifestyle	Improvement in NAS in 68.4% of patients. A decrease in the NAS ≥2 points achieved in 57.9% of patients, and significant average weight loss at the end of intervention (8.3%). Reduction of fibrosis stage in 58.3% of patients when the analysis was limited to patients with stage F2/3 fibrosis.
Stine et al^5^ Noom Weight (NW) mobile app 2023 Single-center RCT	20 patients with MASH (ultrasound, CT or MRI study showing hepatic steatosis and one of the following: FIB-4 ≥1.45 or vibration-controlled transient elastography measurement of liver stiffness>8.2 kPA or FAST score >0.35) and 20 controls	To assess clinically significant body weight loss To assess feasibility (weekly app engagement), acceptability (>50% approached enrolled), and safety of the app	NW mobile app self-monitoring and feedback features for food, exercise, and body weight Digital access to a 1:1 behavior change coach, a support group facilitated by a health coach, and a curriculum All participants were provided with an electronic scale with Bluetooth capabilities	70% of subjects in the NW arm met the feasibility criteria. Significantly decreased body weight with NW compared to standard clinical care (−5.5 kg vs. −0.3 kg, *p*=0.008, −5.4% vs. −0.4%, *p*=0.004).
Mellinger et al^6^ Single-center RCT	30 patients with ALD and 30 controls	To assess feasibility (recruitment and retention rates), and acceptability To assess AUD treatment engagement and alcohol use at 3 and 6 mo	Online behavioral intervention/mobile app Online web application consisting of 2 modules (misconception correction module and treatment preference matching module) designed on principles of the Health Belief Model and the Health Action Process Approach conceptual model of behavior change	Feasible (recruitment rate: 46%, retention rate: 65% at 6 mo, acceptable>90%). Increased AUD treatment engagement at 6 mo (27.3% vs 13.3%, OR=2.3, CI: 0.61–8.76). Trend toward a one-level or greater reduction in WHO drinking levels in the intervention group (OR: 2.25, 95% CI: 0.51–9.97).
Kazankov et al^7^ CirrhoCare app Prospective, observational	20 patients with decompensated cirrhosis and 20 contemporaneous matched controls with standard of care	To assess feasibility and patient engagement To facilitate early detection of new deterioration To identify patients at risk of further decompensation	Daily patient data input and communication with a hepatologist (heart rate, blood pressure, weight, % body water, cognitive function, self-reported well-being, intake of food, fluid, and alcohol)	CirrhoCare app had high patient engagement (≥2 measurements/week) in 85% of the patients and positive user feedback. CirrhoCare app-managed individuals had fewer and shorter readmissions, markedly reduced unplanned paracentesis, and improvement in disease severity scores compared to controls.
Duarte-Rojo et al^8^ EL-FIT (Exercise and Liver FITness) app Prospective, observational	28 patients with cirrhosis listed or undergoing evaluation for LT	To assess feasibility of EL-FIT among candidates of LT To investigate the adequacy of EL-FIT app training-level prescriptions and data transfer fidelity from the tracker to the EL-FIT app database. To assess participant and caregiver perspectives and technology acceptability	Mobile app and physical activity tracker App included a stratification algorithm facilitating exercise prescriptions at proper training levels with educational and exercise/workout videos of corresponding intensity, and collects daily steps, heart rate, sleep time. Gamification features within the app (leaderboard, badges for accomplishments etc.) Involvement of an exercise professional with push notifications and review of participants’ achievements	Patients with cirrhosis were able to use and interact with EL-FIT app (77% had watched at least 1 video, 69% completed at least 1 video section, each with 4-7 videos). Level of training assigned by the EL-FIT app agreed with that from a physical therapist in 89% of cases. 35% of the participants had significant increases in their physical performances based on heart rate–validated steps as a marker of performance.
Lim et al^9^ Nutritionist Buddy (nBuddy) app Parallel RCT	108 patients with MASLD confirmed by steatosis on imaging and BMI ≥23 kg/m^2^ (55 intervention group, 53 control group)	To evaluate the effect of a lifestyle intervention consisting of diet and physical activity enabled by a mobile app and health care professional in facilitating weight loss and improving relevant health indicators in patients with MAFLD	53 controls received standard care with dietary and lifestyle advice by a trained nurse, and intervention group using the nBuddy mobile app in addition to receiving dietary and lifestyle advice by a dietitian via face-to-face session in clinic followed by remote support through mobile app Food diary log, caloric goals, recording of daily steps, manual log of physical activities, weight logging, dietitian access to diet, physical activity, and weight for real-time feedback and encouragement	Intervention group had a 5-fold higher likelihood of achieving ≥5% weight loss compared to the control group at 6 mo. Greater reductions in weight, waist circumference, systolic blood pressure, diastolic blood pressure, ALT, and AST at 6 mo.
Bloom et al^10^ Prospective, observational	25 patients with cirrhosis and ascites	To assess the feasibility of a smartphone app to manage outpatient ascites	Bluetooth-connected scale, PGHD Connect app Transmission of daily weight data to EMR and alerting physician if weight change ≥5 lbs in 1 wk	App was feasible (received weight data into EMR on ≥50% of the days enrolled and providers responded ≥50% of the weight alerts). Transmission of weight data into EMR successfully occurred on 71.2% of study enrollment days. A total of 17 patient readmissions occurred during the study period, with only 4 (24%) related to ascites.

Abbreviations: ALD, alcohol-associated liver disease; ALT, alanine aminotransferase; AST, aspartate aminotransferase; AUD, alcohol-use disorder; BM, bowel movement; BMI, body mass index; BSS, Bristol Stool Scale; EMA, ecological momentary assessment; EMR, electronical medical record; FAST, FibroScan-AST; FIB-4, Fibrosis-4 Index; LT, liver transplantation; MASLD, metabolic dysfunction–associated steatotic liver disease; MASH, metabolic dysfunction–associated steatohepatitis; NAS, nonalcoholic fatty liver disease activity score; RCT, randomized controlled trial; WHO, World Health Organization.

## THE MOBILE APP ERA MATURES

Recently, 3 studies conducted with the aim to identify the utility and benefits of mobile apps in different patient populations with CLD were published in *Hepatology Communications*.

### Digital phenotyping

Wu et al^3^ conducted a study by using AWARE application with the aim to understand the digital phenotypes of patients with alcohol-associated liver disease (ALD) by the collection of passive smartphone sensor data through users’ daily routine activities and delivery of ecological momentary assessments for active collection of data on craving, alcohol, or substance use, and mood in 24 patients with ALD and alcohol-use disorder (AUD). The goal in this study was to assess if smartphone sensors could potentially be used as markers for alcohol, craving, mood, and could predict clinical outcomes such as relapse or readmission. AWARE app used both passive continuous sensor data, meaning without active user engagement, and active data with prompted surveys (alcohol craving and mood assessments). The study concluded that smartphone sensors may serve as proxies for behavioral characteristics and categorize patients into digital phenotypes, such as those at risk of relapse and progression of liver disease. By using ecological momentary assessments and smartphone sensors (accelerometer, applications used, Bluetooth engagement, calls made and duration, text messages sent and length, locations, etc.) as surrogates for alcohol craving to identify patients at risk for relapse, the investigators have created a framework for just-in-time interventions.

### Harm reduction

A randomized controlled trial conducted by Mellinger et al^6^ enrolled 60 patients with ALD with untreated AUD. It was the first behavioral mobile health intervention specifically for AUD treatment in patients with ALD. Half received a novel behavioral intervention delivered via mobile app. Intervention consisted of a single-session online web application with 2 modules (misconception correction module to evaluate the presence of misconceptions of alcohol use, AUD treatment, and liver disease, and treatment preference matching module to assess participants’ rating of treatment preferences on 17 different components of AUD treatment). Outcomes and measures were collected at month 3 and 6 postintervention. It was feasible (recruitment rate: 46%, retention rate: 65% at 6 months, acceptable>90%). It was also effective. The intervention increased AUD treatment engagement rates at 6 months in the intervention group (27.3%) compared to the control group (13.3%), and a trend toward a one-level or greater reduction in WHO (World Health Organization) drinking levels (OR: 2.25, 95% CI: 0.51–9.97).

### Lifestyle change

Stine et al^5^ conducted a 16-week randomized controlled trial, including 20 patients with metabolic dysfunction–associated steatohepatitis (either liver biopsy evidence of metabolic dysfunction–associated steatohepatitis or an ultrasound, CT, or MRI study showing hepatic steatosis and one of the following: Fibrosis-4 Index ≥1.45 or vibration-controlled transient elastography measurement of liver stiffness >8.2 kPA or FibroScan-AST score >0.35) by using Noom app or 20 controls with the standard of care. Noom is a self-management app that guides calorie consumption, exercise, and stress relief. This study was the first to assess a mobile app–delivered lifestyle intervention program in patients with metabolic dysfunction–associated steatohepatitis. In the Noom arm, more patients achieved a clinically significant weight loss of ≥5% of body weight (45% vs. 15%, *p*=0.038), and 70% of the patients met the feasibility criteria (at least once weekly app engagement). The study also suggested that the mobile app may create self-regulatory skills which are considered essential to facilitate sustained weight loss.

## PITFALLS

Despite the potentials for improving care in CLD, mobile apps have several pitfalls that may limit their use. Patients without reliable access to smartphones or the internet would not be able to use the mobile apps. Acceptance, willingness, motivation, consistent and/or meaningful usage of the apps, limited utility of the apps in the setting of cognitive impairments, potential language barrier, concerns for privacy, and data sharing remain some of the limitations with the mobile apps. It is unclear whether those who agree to use mobile apps are representative of contemporary patients with CLD who may have limited access to Wi-Fi, smartphones, or limited comfort with technology. Additionally, imitations across the existing studies are the relatively small patient sizes given the pilot studies, suboptimal patient retention rates, and data transmission inconsistencies or errors with apps.

## THE PATH FORWARD

These studies reveal the future of apps—point-of-care, just-in-time behavioral interventions that can be customized to the patient’s needs, maximizing effectiveness through digital phenotyping.^3,5,6^ Pilot studies should now give way to larger, multicenter evaluations designed for generalizable effects. Randomization, thoughtful control arms, and enrollment of sufficient power to detect meaningful effects are needed. Recruitment methods must be designed for inclusivity, support for enrollment, including download and set-up, are needed, and technological support should be provided. Mobile apps should ideally be acceptable to patients with limited literacy, easy to use, and allow two-sided communications between patient/caregiver and provider. Efforts to anticipate and reduce the need for clinical monitoring of patient inputs while preserving safety are essential. Studies evaluating the specific features of apps (reminders or notifications at specific times, self-tracking, feedback, and individualized recommendations), resulting in behavioral changes are important to identify gaps of knowledge in the understanding of their effectiveness on outcomes of CLD. However, interventions are needed above all. If interventions are multifactorial and conditional on variable patient inputs, sophisticated designs that incorporate the assessment of multiple interventions, such as “SMART” or fractional factorial designs, are needed.

Mobile apps fill multiple important needs in the management of CLD. Our approach to them needs to be deliberate, sophisticated, and generalizable.
